# Endothelial Sestrin2 Coordinates Multiple Protective Pathways to Maintain Angiogenic Function in Diabetes-Associated Endothelial Dysfunction

**DOI:** 10.3390/ijms262311396

**Published:** 2025-11-25

**Authors:** Muhammad Ammar Zahid, Aijaz Parray, Hassaan Anwer Rathore, Abbas Khan, Abdelali Agouni

**Affiliations:** 1 Department of Pharmaceutical Sciences, College of Pharmacy, QU Health, Qatar University, Doha P.O. Box 2713, Qatarhrathore@qu.edu.qa (H.A.R.); abbas.khan@qu.edu.qa (A.K.); 2 The Neuroscience Institute, Academic Health System, Hamad Medical Corporation, Doha P.O. Box 3050, Qatar; aparray@hamad.qa

**Keywords:** Sestrin2, oxidative stress, angiogenesis, diabetes, methylglyoxal, endothelial dysfunction, cardiovascular disease

## Abstract

Diabetes mellitus is prevalent worldwide, with vascular complications responsible for over 70% of deaths associated with the condition. Methylglyoxal (MGO), a by-product of glycolysis, is a significant modulator of vascular dysfunction in diabetes. Sestrin2 (SESN2) has been recognized as a vital regulator of cellular homeostasis and stress responses. Although SESN2’s role in cellular defense is gaining recognition, its precise function in endothelial cells under diabetic-like conditions remains poorly understood. This study examines the role of SESN2 in preserving endothelial cell angiogenic function under MGO-induced stress. The study reveals that SESN2 is a vital regulator of multiple protective pathways, as demonstrated by both loss-of-function and gain-of-function approaches in EA.hy926 endothelial cells. Our data showed that *SESN2* overexpression significantly maintained tubular network formation, proliferation, and invasive capacity under MGO stress, whereas *SESN2* silencing exacerbated MGO-induced impairment of angiogenic capacity. SESN2 was identified as orchestrating NRF2/HO-1 antioxidant pathway activation while simultaneously enhancing VEGF-C expression, offering a dual strategy for cellular protection and angiogenesis. Moreover, SESN2 facilitated a regulated equilibrium of the AKT/mTOR signaling pathway, ensuring synchronized activation during stress conditions. SESN2 also regulated stress-activated MAPK pathways, diminishing P38 and ERK1/2 activation upon MGO exposure. This study highlights SESN2 as a pivotal regulator of endothelial cell homeostasis and angiogenic activity under MGO-induced stress, indicating its potential as a therapeutic target for addressing diabetic vascular complications and improving patient outcomes.

## 1. Introduction

Diabetes mellitus affects over 537 million adults globally, with projections suggesting this number will rise to 783 million by 2045 [1]. Vascular complications remain the primary cause of diabetes-related morbidity and mortality, accounting for approximately 70% of deaths in diabetic patients [2]. These complications arise from complex pathophysiological mechanisms, among which the accumulation of advanced glycation end products (AGEs) and their precursors plays a central role [3]. Methylglyoxal (MGO), a highly reactive α-dicarbonyl compound formed as a by-product of glycolysis, emerges as a key mediator of vascular dysfunction in diabetes [4]. Under hyperglycemic conditions, MGO levels can increase, leading to extensive protein modifications and cellular dysfunction [5].

The endothelium, as the primary interface between blood and tissues, is particularly vulnerable to MGO-induced damage. MGO modifies proteins by forming AGEs, disrupts mitochondrial function, increases oxidative stress, and impairs cellular signaling pathways crucial for endothelial homeostasis [4,6,7]. These perturbations manifest as reduced nitric oxide (NO) bioavailability, increased expression of inflammatory markers, and compromised angiogenic responses [8]. While several protective mechanisms exist to counteract MGO toxicity, including the glyoxalase system and various stress–response proteins, their effectiveness is often overwhelmed in chronic diabetic conditions.

Sestrins (SESNs), a protein family comprising three members (SESN1, SESN2, and SESN3), are highly conserved stress-inducible proteins that have emerged as critical regulators of cellular homeostasis. Recent discoveries have dramatically expanded our understanding of SESN2’s multifaceted roles in cellular protection and metabolic regulation [9]. Beyond its initially characterized functions as a leucine sensor and mammalian target of rapamycin complex 1 (mTORC1) regulator, SESN2 has been identified as a crucial mediator of mitophagy through its direct interaction with Unc-51-like kinase 1 (ULK1) and p62, ensuring the selective removal of damaged mitochondria [10,11]. Furthermore, recent studies have revealed that SESN2 is involved in cellular iron homeostasis, regulating ferroptosis sensitivity by controlling Glutathione peroxidase 4 (GPX4) activity and iron metabolism [12,13].

SESN2 has also emerged as a key player in metabolic reprogramming under stress conditions. Research has demonstrated its role in maintaining metabolic flexibility by regulating AMP-activated protein kinase (AMPK)-dependent glucose uptake and fatty acid oxidation [14,15]. In addition, SESN2 has been found to interact with the Kelch-like ECH-associated protein 1 (KEAP1)/Nuclear factor erythroid 2-related factor 2 (NRF2) pathway in a novel manner, where it not only enhances NRF2 activation but also regulates the transcription of specific antioxidant genes through direct protein–protein interactions [16]. These findings have established SESN2 as a central coordinator of cellular stress responses, linking oxidative stress defense, metabolic regulation, and organelle quality control.

Particularly relevant to vascular function, recent studies have uncovered SESN2’s involvement in endothelial cell protection and its response to ischemic injury. The protein has been shown to mediate endothelial progenitor cell adaptation to stress and increased angiogenesis in response to ischemia [16,17]. Angiogenesis, the formation of new blood vessels from pre-existing vasculature, represents a critical process in tissue repair and regeneration [18]. This complex phenomenon requires coordinated endothelial cell proliferation, migration, and tubulation, all of which are significantly impaired in diabetes [19]. The mechanisms underlying diabetic angiogenic dysfunction are multifaceted, involving alterations in growth factor signaling, increased oxidative stress, and disrupted metabolic homeostasis [20]. Understanding how these pathways intersect and identifying key regulatory molecules is crucial for developing targeted therapeutic strategies.

While recent discoveries highlight SESN2’s importance in cellular homeostasis and stress response, its specific function in endothelial cells under diabetic-like conditions remains poorly understood. The present study investigates how SESN2 modulates endothelial cell function and angiogenic responses in the context of MGO-induced stress, using both loss-of-function and gain-of-function approaches. By examining the molecular mechanisms by which SESN2 influences endothelial cell behavior under diabetic-like conditions, this research aims to identify new therapeutic targets to manage diabetic vascular complications and improve patient outcomes.

## 2. Results

### 2.1. Validation of Experimental Model and SESN2 Modulation

**Figure 1:**
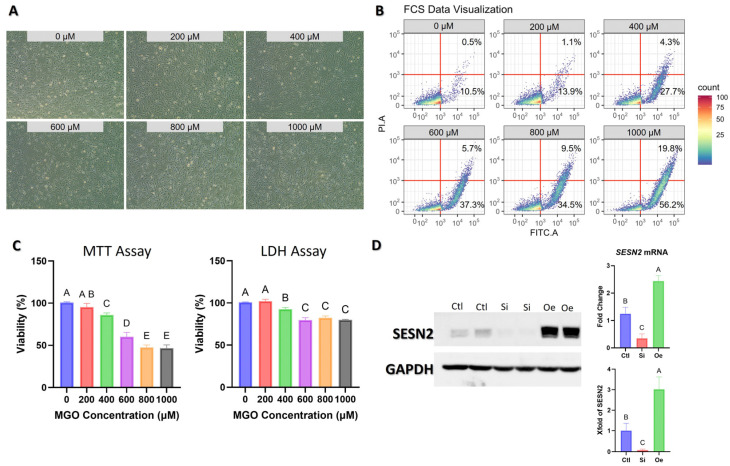
Dose–response analysis of methylglyoxal (MGO) on EA.hy926 endothelial cells and validation of Sestrin2 (SESN2) modulation. (**A**) Representative phase-contrast microscopy images of EA.hy926 cells treated with increasing concentrations of MGO (0–1000 µM) for 18 h. (**B**) Flow cytometry analysis of cells stained with Propidium Iodide (PI) and FITC-Annexin V after MGO treatment. The lower-right quadrant represents early apoptotic cells, while the upper-right quadrant represents late apoptotic/necrotic cells. Percentages indicate the proportion of cells in these quadrants. (**C**) Quantitative analysis of cell viability via MTT assay (left) and cytotoxicity via LDH assay (right) after 18 h of MGO treatment. (**D**) Validation of *SESN2* gene silencing (Si) and overexpression (Oe) 48 h post-transfection. Left: Representative Western blot showing SESN2 protein levels, with GAPDH as a loading control. Right: Relative *SESN2* mRNA expression measured by qRT-PCR (top) and quantification of SESN2 protein levels from Western blots (bottom), normalized to GAPDH. Data are presented as mean ± SD (*n* = 3). Groups that do not share a common letter are significantly different from one another (*p* < 0.05).

Prior to functional assays, we established and validated our experimental model. First, we determined an appropriate concentration of MGO to induce endothelial stress. As detailed in Figure 1A–C, a dose of 600 µM MGO was chosen because it consistently induced morphological signs of stress and significantly reduced cell viability without causing overwhelming cytotoxicity. Second, we confirmed the efficacy of our *SESN2* overexpression and silencing procedures. As shown in Figure 1D, transfection with *SESN2*-targeting small interfering RNA (siRNA) led to a significant decrease in both *SESN2* mRNA and protein levels. In contrast, transfection with a *SESN2* expression plasmid resulted in a robust increase. These validation steps confirm the reliability of our model for the subsequent mechanistic analyses.

### 2.2. SESN2 Expression Levels Modulate Endothelial Cell Tube Formation Capacity

To investigate the role of SESN2 in angiogenesis and endothelial health, we performed endothelial cell tube formation assays and assessed endothelial nitric oxide synthase (*eNOS/NOS3*) mRNA expression under varying SESN2 conditions. Endothelial cells were subjected to three different SESN2 conditions: control (Ctl), *SESN2* silencing (Si), and *SESN2* overexpression (Oe). Additionally, we examined the impact of MGO stress by treating cells with 600 μM MGO for 18 h.

**Figure 2:**
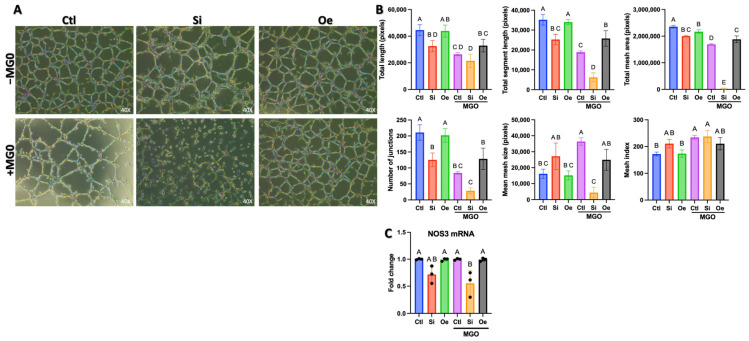
Effect of MGO treatment on endothelial cell tube formation capacity and *eNOS* mRNA expression under SESN2 modulation. (**A**) Representative phase-contrast microscopy images of the endothelial tube formation assay showing vascular network formation across three conditions (Ctl: Control, Si: *SESN2* silencing, Oe: *SESN2* overexpression) in the absence (−MGO, top row) and presence (+MGO, bottom row) of 600 μM MGO treatment. Images were captured 18 h after EA.hy926 endothelial cells were seeded on Matrigel. (**B**) Quantitative analyses of angiogenic parameters, including total tube length (top left), total branching points (top middle), total mesh area (top right), number of junctions (bottom left), mean mesh size (bottom middle), and total number of meshes (bottom right). (**C**) Relative *eNOS (NOS3)* mRNA expression levels measured by qRT-PCR. Data were normalized to GAPDH expression and are presented as fold change relative to the control (−MGO) group, showing the impact of SESN2 modulation and MGO treatment on this key endothelial function gene. Data in panels B and C are presented as mean ± SD (*n* = 3 independent experiments). Groups that do not share a common letter are significantly different from one another (*p* < 0.05).

Phase-contrast microscopy revealed distinct patterns of vascular network formation across different experimental conditions (Figure 2A). In the absence of MGO treatment (−MGO), control cells formed well-organized tubular networks. *SESN2* silencing resulted in a significant reduction in several parameters of network formation (e.g., total tube length; *p* < 0.05 vs. Ctl −MGO), whereas *SESN2* overexpression maintained robust tubular structures comparable to those in control conditions. Upon MGO treatment (+MGO), control cells showed marked impairment in their tube formation capacity. This MGO-induced disruption was notably exacerbated in *SESN2*-silenced cells, whereas *SESN2*-overexpressing cells demonstrated remarkable resistance to MGO-mediated network disruption.

Quantitative analysis of multiple angiogenic parameters revealed significant differences across experimental conditions (Figure 2B). In the absence of MGO, total tube length measurements showed that *SESN2* overexpression maintained levels comparable to those of control (*p* > 0.05), whereas *SESN2* silencing led to a significant decrease. MGO treatment caused a substantial reduction in tube length across all groups; however, *SESN2* overexpression provided partial protection against this effect (*p* < 0.05). The analysis of branching points and mesh formation parameters further supported these findings. Total branching points and mesh area measurements demonstrated that *SESN2* overexpression preserved network complexity under MGO treatment, while *SESN2* silencing exacerbated MGO-induced network simplification. The number of junctions, mean mesh size, and total number of meshes showed similar patterns, with *SESN2* overexpression consistently demonstrating protective effects against MGO-induced impairment of angiogenic capacity.

In parallel, we assessed *eNOS* (*NOS3*) mRNA levels, a critical indicator of endothelial function (Figure 2C). Consistent with the impaired angiogenic capacity, *SESN2* silencing (Si −MGO) significantly reduced *eNOS* mRNA expression compared to control cells. This reduction in *eNOS* mRNA was also observed in *SESN2*-silenced cells treated with MGO (Si +MGO). Interestingly, under the conditions tested, *eNOS* mRNA levels in *SESN2* overexpressing cells (Oe −MGO and Oe +MGO) and control cells treated with MGO (Ctl +MGO) did not show significant changes compared to the untreated control group (Ctl −MGO). This suggests that the severe angiogenic impairment observed in *SESN2*-silenced cells may be partly associated with a compromised eNOS system at the transcript level. While *SESN2* overexpression preserved angiogenic function, particularly under MGO stress, this protective effect, based on these *eNOS* mRNA findings, might primarily involve mechanisms beyond upregulating *eNOS* transcription or preserving basal eNOS functionality that MGO would further impair.

### 2.3. SESN2 Regulates Multiple Aspects of Endothelial Cell Function Under Normal and Stress Conditions

Following our observations of SESN2’s role in tube formation, we investigated whether these effects were mediated through changes in cellular proliferation, invasion capacity, or MMP activity. These analyses helped distinguish between direct effects on angiogenic network formation versus indirect effects through altered cell behavior.

**Figure 3:**
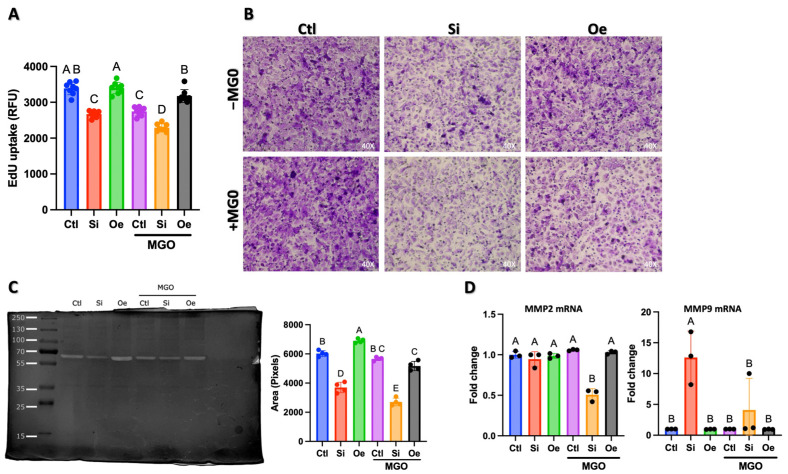
Effects of SESN2 modulation and MGO treatment on endothelial cell proliferation, invasion, MMP activity, and *MMP* mRNA expression. (**A**) Cell proliferation measured by EdU uptake assay (2 h pulse) showing differential proliferative capacity across control (Ctl), *SESN2* silencing (Si), and *SESN2* overexpression (Oe) conditions, with and without 600 μM MGO treatment. Data presented as relative fluorescence units (RFU). (**B**) Representative images of Boyden chamber invasion assay showing EA.hy926 cell invasion through Matrigel-coated membranes after 24 h in the absence (−MGO, top row) and presence (+MGO, bottom row) of MGO treatment. Purple staining indicates invaded cells. (**C**) Representative gelatin zymography demonstrating MMP2 activity in conditioned media across experimental conditions. Active MMP-9 was not detected by zymography under any of the tested conditions. Quantitative analyses of invasion assay (right) and MMP-2 zymographic band intensity (left). (**D**) Relative mRNA expression levels of *MMP2* and *MMP9* measured by qRT-PCR. Data were normalized to GAPDH expression and are presented as fold change relative to the control (−MGO) group. Data in panels A, C, and D are presented as mean ± SD (*n* = 3 independent experiments). Groups that do not share a common letter are significantly different from one another (*p* < 0.05).

Cell proliferation, assessed via 5-Ethynyl-2´-deoxyuridine (EdU) uptake assay, revealed significant differences across SESN2 expression conditions (Figure 3A). Under normal conditions (−MGO), cells showed robust proliferation (3500 RFU), while *SESN2* silencing reduced proliferative capacity by approximately 25% (*p* < 0.05). Notably, *SESN2* overexpression maintained proliferation rates comparable to control conditions. Upon MGO treatment, all groups showed decreased proliferation. However, *SESN2* overexpression provided partial protection against the MGO-induced decline in proliferation, maintaining significantly higher proliferation rates than in the control or silenced conditions (*p* < 0.05).

To determine whether the observed effects on tube formation were solely due to altered proliferation or involved changes in invasive capacity, we performed Boyden chamber invasion assays (Figure 3B). The visual assessment of invaded cells revealed striking differences across conditions. Under normal conditions, *SESN2* silencing markedly reduced invasion capacity compared to control cells, while *SESN2* overexpression maintained invasive potential. This pattern became more pronounced under MGO treatment, with *SESN2*-silenced cells showing minimal invasion, while *SESN2*-overexpressing cells maintained substantial invasive capacity despite MGO stress. Quantitative analysis of invasion (Figure 3C, right panel) supported these observations, showing significant differences between groups that parallel but are distinct from the proliferation patterns. This suggests that SESN2’s effects on endothelial cell function extend beyond merely regulating proliferation.

To investigate the molecular mechanisms underlying these invasion differences, we examined matrix metalloproteinase-2 (MMP-2) activity by gelatin zymography (Figure 3C) and *MMP2* and *MMP9* mRNA expression (Figure 3D). The zymographic analysis revealed active MMP-2 bands across all conditions, while active MMP-9 protein was not detected by this method. Quantification of MMP-2 zymographic activity (Figure 3C, left) showed that *SESN2* overexpression significantly enhanced MMP-2 activity under both normal and MGO-stressed conditions, while *SESN2* silencing reduced it. This pattern of MMP2 activity closely mirrors the invasion results.

Analysis of mRNA expression revealed more complex regulation (Figure 3D). *MMP2* mRNA levels were significantly decreased only in the *SESN2*-silenced cells under MGO stress (Si +MGO), suggesting that MGO stress in the absence of SESN2 specifically downregulates *MMP2* transcription, which aligns with the observed reduction in its activity in this group. In other conditions, *MMP2* mRNA levels were not significantly altered, suggesting that the increased MMP2 activity observed with *SESN2* overexpression may involve post-transcriptional mechanisms or enhanced protein stability/activation. Interestingly, *MMP9* mRNA, whose active protein was not detected by zymography, showed significant upregulation in *SESN2*-silenced cells under basal conditions (Si − MGO). The functional relevance of this *MMP9* mRNA upregulation in the absence of detectable active protein remains to be investigated. Still, it suggests a potential dysregulation of matrix-remodeling gene expression when *SESN2* is silenced. Overall, these findings indicate that SESN2 regulates invasive capacity by modulating MMP-2 activity, with transcriptional control of *MMP-2* becoming particularly evident under combined SESN2 deficiency and MGO stress.

### 2.4. SESN2 Promotes Angiogenesis Through Parallel Activation of NRF2/HO-1 Antioxidant Pathway and VEGF-C Expression

**Figure 4:**
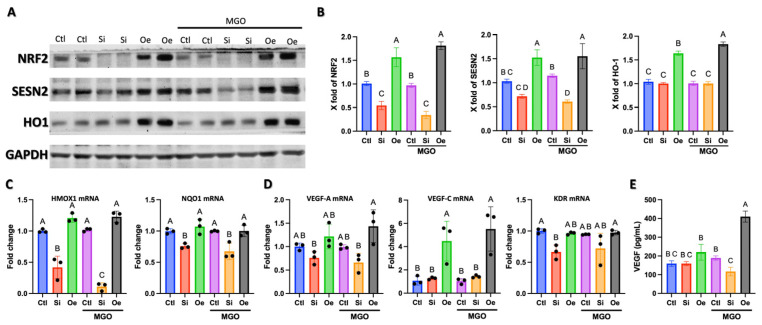
SESN2 modulates the NRF2/HO-1 antioxidant axis and the expression of key angiogenic factors at both protein and mRNA levels. (**A**) Representative Western blot analysis showing protein expression of NRF2, SESN2, and HO-1, with GAPDH as loading control. Cells were treated under control conditions (Ctl), SESN2 silencing (Si), or SESN2 overexpression (Oe), with or without 600 μM MGO treatment for 18 h. Blots shown are representative of *n* = 3 independent experiments. (**B**) Quantitative analysis of NRF2/GAPDH, SESN2/GAPDH, and HO-1/GAPDH protein expression. (**C**) Relative mRNA expression levels of NRF2 target genes, *HMOX1* and *NQO1*, measured by qRT-PCR. (**D**) Relative mRNA expression levels of key angiogenic factors, *VEGFA*, *VEGFC*, and their receptor KDR (*VEGFR2*), measured by qRT-PCR. For panels C and D, mRNA data were normalized to GAPDH expression and are presented as fold change relative to the control (−MGO) group. (**E**) Quantification of secreted VEGF-C protein in conditioned media measured by ELISA. All quantitative data (panels (**B**–**E**)) are presented as mean ± SD (*n* = 3–5 independent experiments). Groups that do not share a common letter are significantly different from one another (*p* < 0.05).

While our previous results established that SESN2 regulates multiple endothelial cell functions critical for angiogenesis, we further investigated the underlying mechanisms. The NRF2/HO-1 antioxidant pathway is crucial for regulating angiogenesis and could provide additional mechanistic insight into SESN2’s protective effects under oxidative stress. Western blot analysis revealed coordinated regulation of NRF2, SESN2, and HO-1 protein levels across different experimental conditions (Figure 4A). Under basal conditions, *SESN2* silencing resulted in a 50% reduction in NRF2 protein levels (*p* < 0.05) and a corresponding decrease in HO-1 expression. Conversely, *SESN2* overexpression significantly increased NRF2 and HO-1 protein levels (1.5-fold, *p* < 0.05), establishing a direct relationship between SESN2 and the NRF2/HO-1 antioxidant axis. The regulatory impact of SESN2 on the NRF2/HO-1 pathway became more pronounced under MGO-induced oxidative stress. Quantitative analysis (Figure 4B) showed that, while control cells exhibited a modest reduction in NRF2 and HO-1 levels under MGO treatment, *SESN2*-overexpressing cells maintained significantly elevated levels of both proteins (approximately 1.8-fold higher than in control cells, *p* < 0.05). This maintenance of antioxidant pathway activation aligns with the preserved angiogenic capacity observed in tube formation assays under oxidative stress. *SESN2* overexpression led to a substantial increase in *VEGFC* mRNA levels (4- to 6-fold, *p* < 0.05), particularly under MGO stress (Figure 4D). To determine whether this transcriptional upregulation led to increased protein secretion, we measured VEGF-C levels in conditioned media by enzyme-linked immunosorbent assay (ELISA). In line with the mRNA data, *SESN2* overexpression increased secreted VEGF-C levels, a protective effect that was maintained under MGO stress (Figure 4E). This enhanced VEGF-C expression provides a direct mechanistic link supporting the improved angiogenic capacity observed in SESN2-overexpressing cells.

### 2.5. SESN2 Orchestrates Angiogenic Response Through Differential Regulation of AKT/mTOR Signaling

**Figure 5:**
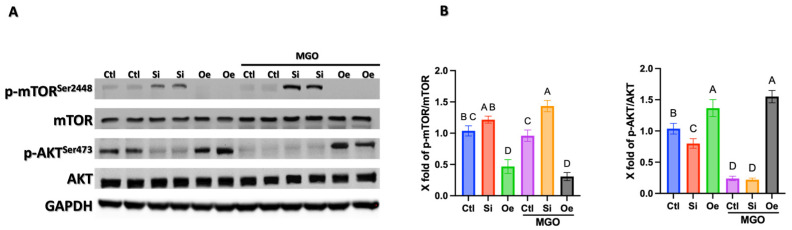
SESN2 differentially regulates the AKT/mTOR signaling pathway in endothelial cells under normal and MGO-stressed conditions. (**A**) Representative Western blot analysis showing protein expression of phosphorylated AKT (p-AKT Ser473), total AKT, phosphorylated mTOR (p-mTOR Ser2448), total mTOR, with GAPDH as a loading control. Endothelial cells were subjected to control (Ctl), SESN2 silencing (Si), or SESN2 overexpression (Oe) conditions, with or without 600 μM MGO treatment for 18 h. Blots shown are representative of *n* = 3 independent experiments. (**B**) Quantitative analysis of p-AKT/total AKT ratio (left) and p-mTOR/total mTOR ratio (right). Data are presented as mean ± SD (*n* = 3 independent experiments). Groups that do not share a common letter are significantly different from one another (*p* < 0.05).

To elucidate the molecular mechanisms underlying SESN2-mediated regulation of endothelial cell function, we examined the AKT/mTOR signaling axis, another important pathway in angiogenesis regulation and metabolic control. Western blot analysis revealed distinct patterns of AKT and mTOR phosphorylation across different SESN2 expression conditions, both under normal and MGO-stressed states (Figure 5A). Under basal conditions, *SESN2* silencing led to a paradoxical response in the AKT/mTOR pathway: decreased AKT phosphorylation (Ser473) while simultaneously increasing mTOR phosphorylation (Ser2448). Conversely, *SESN2* overexpression maintained balanced activation of both pathways, with moderately elevated AKT phosphorylation while keeping mTOR phosphorylation low compared to control conditions. The divergent regulation became more pronounced under MGO treatment. Quantitative analysis (Figure 5B) revealed that MGO stress significantly suppressed both p-AKT/AKT and p-mTOR/mTOR ratios in control cells. Notably, *SESN2* silencing maintained this effect on AKT phosphorylation while paradoxically increasing mTOR phosphorylation. This dysregulated, uncoordinated signaling pattern between AKT and mTOR in *SESN2*-silenced cells aligns with the impaired angiogenic capacity observed in our functional assays. Remarkably, *SESN2* overexpression maintained significantly higher p-AKT/AKT ratios under MGO stress (approximately 1.5-fold higher than control, *p* < 0.05), while preserving reduced mTOR activation. This maintenance of coordinated AKT/mTOR signaling under oxidative stress provides a mechanistic explanation for the preserved angiogenic capacity observed in our tube formation, proliferation, and invasion assays.

### 2.6. Modulation of the MAPK/ERK Signaling Pathway by SESN2

**Figure 6:**
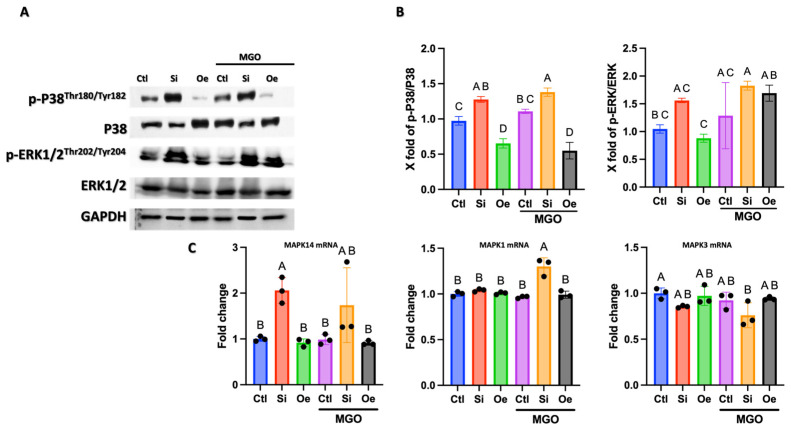
SESN2 modulates stress-activated MAPK signaling pathways and MAPK gene expression in endothelial cells. (**A**) Representative Western blot analysis showing protein expression and phosphorylation status of P38 MAPK and ERK1/2, with GAPDH as loading control. Cells were treated under control conditions (Ctl), SESN2 silencing (Si), or SESN2 overexpression (Oe), with or without 600 μM MGO treatment for 18 h. Blots shown are representative of *n* = 3 independent experiments. (**B**) Quantitative analysis of phosphorylated protein levels normalized to their respective total protein counterparts (p-P38/P38 and p-ERK/ERK ratios). (**C**) Relative mRNA expression levels of *MAPK14 (p38α), MAPK1 (ERK2), and MAPK3 (ERK1),* measured by qRT-PCR. mRNA data were normalized to GAPDH expression and are presented as fold change relative to the control (−MGO) group. All quantitative data (panels (**B**,**C**)) are presented as mean ± SD (*n* = 3 independent experiments). Groups that do not share a common letter are significantly different from one another (*p* < 0.05).

To understand SESN2’s impact on stress-activated signaling, we examined key MAPK pathways at both the protein phosphorylation and mRNA expression levels. Western blot analysis revealed significant modulation of P38 MAPK and ERK1/2 activation by SESN2 in endothelial cells, particularly under MGO stress (Figure 6A,B). *SESN2* silencing (Si) resulted in significantly elevated phosphorylation of P38 (p-P38) compared to control (Ctl), an effect exacerbated by MGO treatment (approximately 1.4-fold increase vs. Ctl + MGO, *p* < 0.05). Conversely, *SESN2* overexpression (Oe) maintained p-P38 at lower levels, even in the presence of MGO, suggesting protection against stress-induced P38 activation. A similar pattern was observed for ERK1/2 phosphorylation (p-ERK1/2), where *SESN2* silencing under MGO stress led to a marked increase (approximately 1.8-fold higher than Ctl + MGO, *p* < 0.05), while *SESN2* overexpression moderated this activation. Total protein levels of P38 and ERK1/2 remained relatively constant (Figure 6A), indicating changes primarily in phosphorylation status.

We then investigated whether these changes in MAPK activation were accompanied by alterations in the mRNA expression of the kinases themselves (Figure 6C). Interestingly, *MAPK14 (p38α)* mRNA levels were significantly increased upon *SESN2* silencing, both in the absence (Si − MGO) and presence of MGO (Si + MGO). This suggests that SESN2 deficiency may lead to compensatory or stress-induced upregulation of *MAPK14* gene expression, potentially increasing the pool of P38 available for phosphorylation. For the ERK pathway components, *MAPK1 (ERK2)* mRNA was significantly upregulated only in the *SESN2*-silenced cells treated with MGO (Si + MGO). *MAPK3 (ERK1)* mRNA levels did not show significant changes across the tested conditions. *SESN2* overexpression did not significantly alter the mRNA levels of these MAPKs compared to control conditions. These findings suggest that, while the primary impact of SESN2 on MAPK signaling appears to be at the level of phosphorylation, *SESN2* deficiency can also lead to upregulation of *MAPK14* and, under MGO stress, *MAPK1* gene expression, which might further sensitize cells to stress in its absence.

### 2.7. Apoptotic Response Pathway Regulation

**Figure 7:**
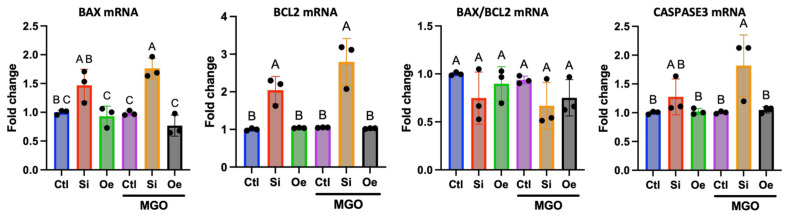
SESN2 modulates the expression of apoptosis-related mRNAs in endothelial cells under MGO stress. Relative mRNA expression levels of *BAX, BCL2*, and *CASP3* (Caspase-3), along with the *BAX/BCL2* mRNA ratio, were measured by qRT-PCR. mRNA data were normalized to *GAPDH* expression and are presented as fold change relative to the control (−MGO) group. All quantitative data are presented as mean ± SD (*n* = 3 independent experiments). Groups that do not share a common letter are significantly different from one another (*p* < 0.05).

To determine SESN2’s role in regulating the apoptotic response, we examined the expression of key apoptosis-related genes (Figure 7). Interestingly, both *BAX* mRNA and *BCL2* mRNA levels were found to be significantly increased upon *SESN2* silencing, both in the absence (Si −MGO) and presence of MGO (Si +MGO). Despite the concomitant increase in both transcripts, the calculated *BAX*/*BCL2* mRNA ratio did not differ significantly across these groups. *CASP3* mRNA levels showed a tendency to increase in *SESN2*-silenced cells under MGO stress (Si +MGO), although this did not reach statistical significance in all replicates. *SESN2* overexpression did not significantly alter mRNA levels of *BAX*, *BCL2*, or *CASP3* compared with control under MGO stress.

## 3. Discussion

The present study reveals SESN2 as a critical orchestrator of endothelial cell function and resilience, particularly under diabetic-like conditions simulated by MGO-induced stress. Our findings, now strengthened by analyses at both protein and mRNA levels, establish multiple interconnected mechanisms through which SESN2 coordinates cellular protection and angiogenic responses. The remarkable preservation of tubular network formation in *SESN2*-overexpressing cells under MGO stress, coupled with the exacerbation of network disruption and impaired *eNOS/NOS3* mRNA expression in *SESN2*-silenced cells (Figure 2), points to SESN2’s fundamental role in maintaining endothelial homeostasis and angiogenic potential. While *eNOS* mRNA levels were notably reduced upon *SESN2* silencing, suggesting a compromised NO system contributing to angiogenic defects, *SESN2* overexpression maintained angiogenic function without significantly upregulating *eNOS* mRNA beyond control levels. This indicates that while basal SESN2 is essential for *eNOS* expression, its protective angiogenic effects under stress may also involve other pathways or post-transcriptional eNOS regulation. This protection extends beyond mere stress resistance, as evidenced by the maintained proliferation rates and invasive capacity in *SESN2*-overexpressing cells under MGO stress (Figure 4).

We modeled diabetic-like vascular injury using MGO, a reactive dicarbonyl and AGE precursor that directly drives endothelial oxidative and inflammatory injury more specifically than high glucose alone. While circulating MGO levels in diabetic plasma are typically in the low micromolar range, supra-physiological concentrations are commonly required in vitro to elicit measurable, acute stress within 18–24 h. Serum proteins and amino acids in culture medium quench a substantial portion of MGO, necessitating higher applied doses. The use of 600 µM MGO in the present study is consistent with established endothelial literature, which demonstrates that concentrations in the high hundreds of micromolar reliably induce dysfunction, reactive oxygen species (ROS) generation, and tight-junction disruption. For example, Lee et al. used 0.6–1.0 mM MGO in Human umbilical vein endothelial cells (HUVECs) and aortic endothelial cells to characterize ROS-dependent suppression of Akt/mTOR and apoptosis [4]. Similarly, Jarisarapurin et al. observed minimal cytotoxicity in EA.hy926 cells below 600 µM, with pronounced cell death only at or above this threshold [21]. Our viability data also show that EA.hy926 cells are comparatively tolerant to the effects of MGO. Wang et al. reported that 200 µM MGO activated the PI3K/Akt–NRF2/HO-1 pathway in HUVECs, directly linking glycative stress to antioxidant defense [22]. Collectively, these findings support 600 µM as a validated, sublethal concentration that reliably engages NRF2-dependent protective mechanisms in EA.hy926 cells without non-specific cytotoxicity—ideal for probing SESN2-mediated regulation under glycative stress.

The regulation of invasive capacity by SESN2 appears to involve complex control of MMPs. While *SESN2* overexpression enhanced MMP2 activity, and silencing reduced it (Figure 3C), *MMP2* mRNA was significantly downregulated only in *SESN2*-silenced cells under MGO stress (Figure 3D). This suggests that under combined SESN2 deficiency and MGO stress, transcriptional suppression of *MMP2* contributes to reduced activity. However, the increased MMP2 activity with *SESN2* overexpression, without a corresponding rise in *MMP2* mRNA, suggests a dominant role for post-transcriptional mechanisms, such as altered protein translation, stability, or pro-MMP2 activation in these conditions. The curious upregulation of *MMP9* mRNA in *SESN2*-silenced cells under basal conditions, despite no detectable active MMP9 protein (Figure 3D), hints at dysregulation of matrix remodeler gene expression upon SESN2 loss, with functional consequences warranting further study.

Our molecular analyses reveal a multi-layered mechanism by which SESN2 maintains endothelial function through robust activation of the NRF2/HO-1 antioxidant pathway and modulation of angiogenic factor expression, now demonstrated at both the protein and transcriptional levels (Figure 4). *SESN2* overexpression not only increased NRF2 and HO-1 protein but also significantly upregulated the mRNA expression of *HMOX1* (encoding HO-1) and another NRF2 target, *NQO1*, especially under MGO stress (Figure 4C). Conversely, *SESN2* silencing reduced these transcripts, solidifying SESN2’s role as a critical upstream regulator of NRF2-mediated transcriptional antioxidant responses. SESN2’s impact on angiogenic signaling parallels this coordinated defense. *SESN2* overexpression substantially increased *VEGFC* mRNA under both basal and MGO conditions, and *VEGFA* mRNA under MGO stress (Figure 4D). The reduction of *KDR (VEGFR2)* mRNA in *SESN2*-silenced cells further implies a compromised VEGF signaling axis. The ability of SESN2 to transcriptionally upregulate key NRF2 antioxidant genes and crucial angiogenic factors, such as VEGF, provides a strong mechanistic basis for its protective and pro-angiogenic effects. Our findings, now confirmed at both the mRNA transcript and secreted protein levels for VEGF-A, demonstrate that SESN2 coordinates a comprehensive, functional pro-angiogenic response under conditions of glycative stress. The role of the NRF2/HO-1 axis in angiogenesis is well-documented, potentially involving decreased oxidative stress, hypoxia-inducible factor-1 (HIF-1) stabilization, PI3K/AKT activation, or increased VEGF expression [23,24]. Previous studies have indeed linked SESN2’s protective effects in ischemia/reperfusion to increased VEGF via NRF2/HO-1 [17] and its role in promoting endothelial progenitor cell functions through the KEAP1/NRF2 pathway [16]. Our detailed mRNA data significantly expand on these by showing that SESN2 exerts broad transcriptional control over this network.

Our molecular analyses also reveal a mechanism by which SESN2 maintains endothelial function by balancing the AKT/mTOR signaling axis (Figure 5). The ability of SESN2 to preserve AKT phosphorylation while preventing aberrant mTOR hyperactivation under oxidative stress represents a key molecular rheostat in endothelial cells. This integration of pro-survival AKT signals and mTOR regulation by SESN2 has been previously shown to protect cells against energetic stress [25], and we have noted increased mTOR activation with *SESN2* silencing alone [26]. This balanced regulation likely explains the preserved angiogenic capacity observed in *SESN2*-overexpressing cells under MGO stress.

Our investigation of MAPK/ERK signaling and apoptotic pathways further illuminates SESN2’s multifaceted role. *SESN2* silencing exacerbated MGO-induced phosphorylation of p38 and ERK1/2 (Figure 6B), which coincided with increased mRNA expression of *MAPK14 (p38α)* under both basal and MGO stress, and *MAPK1 (ERK2)* under MGO stress (Figure 6C). This suggests that SESN2 deficiency not only fails to buffer against stress-induced MAPK activation but may also contribute to a heightened stress response by transcriptionally upregulating these kinase genes, thereby creating a larger pool of activated kinases. Conversely, *SESN2* overexpression maintained lower MAPK activation without significantly altering their baseline mRNA levels, indicating its protective effect here is primarily at the post-transcriptional/activation level. As far as the silencing of *SESN2* is concerned, we previously observed an increase in ERK1/2 phosphorylation in endothelial cells with and without endoplasmic reticulum stress [26]. Interestingly, at the mRNA level, *SESN2* silencing led to increased levels of both pro-apoptotic *BAX* and anti-apoptotic *BCL2* transcripts, though the *BAX*/*BCL2* mRNA ratio remained essentially unchanged. This somewhat paradoxical transcriptional response might reflect a stressed cell’s attempt to trigger apoptosis while simultaneously initiating counteracting survival signals. The tendency for *CASP3* mRNA to increase with *SESN2* silencing under MGO stress further supports a shift towards apoptosis. *SESN2* overexpression, however, does not alter the basal mRNA levels of these apoptotic regulators, again pointing towards crucial post-transcriptional control. Our previous work also demonstrated that SESN2 deficiency exacerbates apoptosis and ROS production and can increase p-ERK1/2 under stress [26], supporting the current findings.

Particularly noteworthy is the complex, multi-layered regulation exerted by SESN2. It not only fine-tunes protein activation and stability but also, as shown by our new mRNA data, influences the transcriptional landscape of crucial antioxidant, angiogenic, MAPK, and apoptotic pathways. The activation of MAPK, evidenced by transcriptional dysregulation of these pathways, suggests that SESN2 acts as a critical checkpoint preventing the hyperactivation of stress–response pathways that might otherwise trigger apoptotic cascades.

It is essential to acknowledge the limitations of our study model. We used the EA.hy926 cell line, a robust tool for mechanistic studies. However, there are recognized differences in gene expression and functional responses between this immortalized cell line and primary endothelial cells, such as HUVECs [27,28]. Therefore, while our findings provide critical insights into the molecular pathways governed by SESN2, direct extrapolation of these results to a clinical setting should be approached with caution. Future validation of our key findings in primary endothelial cells would be a valuable next step to strengthen their physiological relevance.

These findings have significant implications for therapeutic approaches to diabetic vascular complications. The ability of SESN2 to preserve endothelial function through multiple complementary mechanisms, now understood to operate at both transcriptional and post-transcriptional levels, suggests that therapeutic strategies targeting SESN2 might provide more comprehensive protection than approaches targeting individual downstream pathways. The maintained angiogenic capacity and molecular homeostasis in *SESN2*-overexpressing cells under MGO stress remarkably suggest potential applications in diabetic wound healing and other conditions requiring therapeutic angiogenesis.

## 4. Materials and Methods

### 4.1. Chemicals and Reagents

Dulbecco’s Modified Eagle Medium (DMEM), fetal bovine serum (FBS), penicillin–streptomycin (10,000 U/mL), and phosphate-buffered saline (PBS) were purchased from Gibco (Thermo Fisher Scientific, Waltham, MA, USA). MGO (40% solution in water) and dimethyl sulfoxide (DMSO) were obtained from Sigma-Aldrich (St. Louis, MO, USA). Growth factor-reduced Matrigel and BD BioCoat™ Matrigel Invasion Chambers (8 μm pore size) were from BD Biosciences (Franklin Lakes, NJ, USA). Click-iT™ EdU Cell Proliferation Kit was purchased from Thermo Fisher Scientific. Enhanced chemiluminescence (ECL) detection reagent was obtained from Abcam (Cambridge, UK). JetPRIME^®^ transfection reagent and INTERFERin^®^ siRNA transfection reagent were obtained from Polyplus-transfection (Illkirch, France).

### 4.2. Cell Cultures

EA.hy926 endothelial cells (ATCC CRL-2922, ATCC, Manassas, VA, USA) were cultured in DMEM supplemented with 1% penicillin/streptomycin (100 U/mL) and 10% FBS. Cells were maintained at 37 °C in a humidified incubator with 5% CO_2_ atmosphere. Cells were passaged using 0.25% trypsin-EDTA (Gibco) when reaching 80–90% confluence. The EA.hy926 cell line was selected for this study as a well-established and widely used model for investigating endothelial cell biology. Its key advantages include high stability and reproducibility across experiments, which is crucial for minimizing the variability often associated with primary cells from different donors and passages. Furthermore, the high transfection efficiency of EA.hy926 cells is essential for the gain-of-function and loss-of-function experiments that underpin our mechanistic investigation. While this cell line retains critical endothelial characteristics, we acknowledge the known differences in transcriptomic and functional profiles compared to primary endothelial cells. For experimental conditions, MGO was added to the culture medium at specified concentrations 18 h prior to assays. The concentration of MGO (600 µM) used in all experiments was determined based on preliminary dose–response analyses (Figure 1). We assessed cell morphology, apoptosis, viability (MTT assay), and cytotoxicity (LDH assay) across a range of MGO concentrations (0–1000 µM). The 600 µM concentration was selected because it induced a significant level of cellular stress, with approximately 60% viability remaining in the MTT assay (Figure 1C). This dose was optimal for robustly activating cellular stress responses without inducing overwhelming, non-specific cell death, thus providing a suitable model for investigating protective signaling pathways.

### 4.3. Tube Formation Assay

In vitro tube formation assays were performed using growth factor-reduced Matrigel according to established protocols [29]. Matrigel was thawed overnight at 4 °C, and 200 μL was added to each well in 12-well plates. After polymerization at 37 °C for 30 min, EA.hy926 cells (1.4 × 10^5^ cells/well) were seeded onto solidified Matrigel in complete culture medium and monitored for 24 h. Images were captured using an inverted EVOS phase-contrast microscope (Thermo Fisher Scientific, Waltham, MA, USA) equipped with a digital camera. Tube formation was analyzed using the Angiogenesis Analyzer plugin in ImageJ version 1.5.4p (National Institutes of Health, Bethesda, MD, USA), quantifying parameters including total tube length, branch number, and mesh formation [30].

### 4.4. Cell Invasion and Migration

Cell invasion was assessed using BD BioCoat™ Invasion Chambers according to the manufacturer’s instructions. Briefly, 2.5 × 10^4^ cells were seeded in a serum-free medium in the upper chamber, with a complete medium containing 10% FBS as a chemoattractant in the lower chamber. After 24 h, non-invading cells were removed, and invaded cells were fixed with 4% paraformaldehyde and stained with 0.1% crystal violet.

### 4.5. Scratch Migration Assay

For wound healing assays, EA.hy926 monolayers were mechanically disrupted using a sterile 1000 μL pipette tip, washed with PBS, and cultured in a serum-containing medium. Wound closure was monitored after 18 h [31].

### 4.6. Cell Proliferation

Cell proliferation was evaluated using the Click-iT™ EdU assay according to standard protocols. Cells were incubated with 10 μM EdU for 2 h, fixed with 4% paraformaldehyde, and permeabilized with 0.5% Triton X-100. The click reaction was performed according to the manufacturer’s instructions, and fluorescence was measured using Amplex™ UltraRed reagent (Invitrogen, Thermo Fisher Scientific, Waltham, MA, USA). Fluorescence was measured using a microplate reader (Multiskan SkyHigh, Thermo Fisher Scientific, Waltham, MA, USA) at an excitation/emission of 571/585 nm.

### 4.7. Matrix Metalloproteinase Activity

Gelatin zymography was performed using standard protocols to assess Matrix metalloproteinase (MMP) activity in conditioned culture media. Samples were mixed with non-reducing sample buffer and separated on 10% SDS-PAGE containing 0.1% gelatin. Following electrophoresis, gels were washed with 2.5% Triton X-100 to remove SDS and incubated in development buffer (50 mM Tris-HCl, pH 7.5, 200 mM NaCl, 5 mM CaCl_2_, 1 μM ZnCl_2_, 1% Triton X-100) at 37 °C for 18 h. Gels were stained with 0.5% Coomassie Blue and destained to visualize proteolytic activity using a ChemiDoc imaging system (Bio-Rad, Hercules, CA, USA).

### 4.8. Total RNA Isolation, cDNA Synthesis, and Quantitative Real-Time PCR (qPCR)

Total RNA was extracted from EA.hy926 cells using the innuPREP RNA Kit (Analytik Jena, Berlin, Germany) according to the manufacturer’s instructions. The concentration and purity of the isolated RNA were determined using a NanoDrop spectrophotometer (Model 2000, Thermo Fisher Scientific, Waltham, MA, USA).

First-strand complementary DNA (cDNA) was synthesized from 1 μg of total RNA using the RevertAid First Strand cDNA Synthesis Kit (Thermo Fisher Scientific, Waltham, MA, USA) following the manufacturer’s protocol, utilizing random hexamers.

Quantitative real-time PCR (qPCR) was performed on a QuantStudio 5 Real-Time PCR System (Applied Biosystems, Thermo Fisher Scientific, Waltham, MA, USA). Reactions were set up in 10 μL volumes containing 4.5 μL of cDNA template, 0.25 μM of each forward and reverse primer (sequences listed in Table 1), and 1X Luna Universal qPCR Master Mix (New England Biolabs, Ipswich, MA, USA). The thermal cycling conditions were as follows: an initial denaturation step at 95 °C for 1 min, followed by 40 cycles of denaturation at 95 °C for 15 s, annealing at 60 °C for 30 s, and extension at 72 °C for 30 s. A melt curve analysis was performed at the end of each run to confirm the specificity of the amplification product and the absence of primer dimers.

Relative gene expression was quantified using the ΔΔCt method. The cycle threshold (Ct) values for each target gene were normalized to those of the housekeeping gene, Glyceraldehyde-3-phosphate dehydrogenase (GAPDH). Data are expressed as fold change relative to the control group. All qPCR reactions were performed in triplicate for each sample.

### 4.9. Western Blot Analysis

Cells were lysed in radioimmunoprecipitation assay (RIPA) lysis buffer (25 mM Tris-HCl, pH 7.6, 150 mM NaCl, 1% NP-40, 1% sodium deoxycholate, 0.1% SDS) supplemented with a cocktail of Pierce protease and phosphatase inhibitors (Thermo Fisher Scientific, Waltham, MA, USA). Protein concentrations were determined using the Pierce BCA assay (Thermo Fisher Scientific, Waltham, MA, USA). Equal amounts of protein (30 μg) were separated by 10% SDS-PAGE and transferred to a PVDF membrane. Membranes were blocked with 5% non-fat dry milk in TBST for 1 h at room temperature and incubated overnight at 4 °C with primary antibodies: SESN2 (1:1000), NRF2 (1:1000), heme oxygenase HO-1 (1:1000), Akt (1:2000), phospho-Akt (Ser473) (1:1000), mTOR (1:1000), phospho-mTOR (Ser2448) (1:1000), ERK1/2 (1:1000), phospho-ERK1/2 (Thr202/Tyr204) (1:2000), p38 MAPK (1:1000) and phospho-p38 MAPK (Thr180/Tyr182) (1:1000). After washing, membranes were incubated with Horseradish peroxidase (HRP)-conjugated secondary antibodies (Cell Signaling Technology, Danvers, MA, USA) for 1 h at room temperature. Protein bands were visualized using an ECL detection reagent and imaged using a ChemiDoc imaging system (Bio-Rad, Hercules, CA, USA).

### 4.10. VEGF ELISA

The concentration of secreted VEGF-C in the conditioned cell culture media was quantified using a commercially available Human VEGF-C Enzyme-Linked Immunosorbent Assay (ELISA) kit (R&D Systems^®^, Minneapolis, MN, USA; Catalog Number DVE00) according to the manufacturer’s instructions, and concentrations were calculated based on a standard curve.

### 4.11. Gene Manipulation

For overexpression studies, DNA transfections were performed using jetPRIME^®^ reagent (Polyplus-transfection, Illkirch, France) at a 1:1 DNA:reagent ratio in an antibiotic-free medium. For each well of a 6-well plate, 1 μg of plasmid DNA was used. Gene silencing was achieved using siRNA transfection (final concentration 0.75 nM) with INTERFERin^®^ (Polyplus-transfection, Illkirch, France) reagent according to the manufacturer’s protocols. Cells were analyzed 48 h post-transfection. Transfection efficiency was verified by Western blot and qRT-PCR analysis (Figure 1D). These gene manipulation protocols are well established in our laboratory and have been used in previous publications [26,32].

### 4.12. Statistical Analysis

Data are presented as means ± standard deviation (SD) from at least three independent experiments (*n*). Data normality was tested each time using the Shapiro–Wilk test. Statistical comparisons between experimental groups were performed using Student’s *t*-test for two groups.

For normally distributed data, a one-way analysis of variance (ANOVA) followed by Tukey’s post hoc test was applied for multiple comparisons. For non-Gaussian data, the Kruskal–Wallis test, followed by Dunn’s multiple comparisons post hoc test, was used. GraphPad Prism 10.4.0 software (GraphPad Software Inc., San Diego, CA, USA) was used for statistical analyses. *p* values < 0.05 were considered statistically significant.

## 5. Conclusions

In conclusion, this study establishes SESN2 as a crucial regulator of endothelial cell function under MGO stress, demonstrating its orchestration of multiple protective mechanisms to maintain cellular homeostasis and angiogenic capacity. The identification of SESN2’s role in coordinating AKT/mTOR signaling, robustly activating the NRF2/HO-1 pathway at both protein and mRNA levels, modulating MAPK/apoptotic signaling through both kinase activation and gene expression, and influencing the transcriptional profile of key angiogenic factors and eNOS, provides new and deeper insights into endothelial protection under oxidative stress. These findings, significantly enriched by the mRNA expression data, not only advance our understanding of endothelial cell biology but also provide a stronger foundation for developing new therapeutic approaches to address the growing global burden of diabetic vascular complications. Future studies should focus on developing strategies to modulate SESN2 activity in vivo and investigating its potential role in other vascular pathologies.

## Figures and Tables

**Table 1:** Primer sequences used for quantitative real-time PCR.

Gene	Forward Primer (5′-3′)	Reverse Primer (5′-3′)
*MMP2*	TACAGGATCATTGGCTACACACC	GGTCACATCGCTCCAGACT
*MMP9*	TGTACCGCTATGGTTACACTCG	GGCAGGGACAGTTGCTTCT
*NOS3*	TGATGGCGAAGCGAGTGAAG	ACTCATCCATACACAGGACCC
*VEGFA*	AGGGCAGAATCATCACGAAGT	AGGGTCTCGATTGGATGGCA
*VEGFC*	GAGGAGCAGTTACGGTCTGTG	TCCTTTCCTTAGCTGACACTTGT
*KDR*	GGCCCAATAATCAGAGTGGCA	CCAGTGTCATTTCCGATCACTTT
*BAX*	CCCGAGAGGTCTTTTTCCGAG	CCAGCCCATGATGGTTCTGAT
*BCL2*	GGTGGGGTCATGTGTGTGG	CGGTTCAGGTACTCAGTCATCC
*CASP3*	CATGGAAGCGAATCAATGGACT	CTGTACCAGACCGAGATGTCA
*HMOX1*	AAGACTGCGTTCCTGCTCAAC	AAAGCCCTACAGCAACTGTCG
*NQO1*	GAAGAGCACTGATCGTACTGGC	GGATACTGAAAGTTCGCAGGG
*SESN2*	CCTCTGGGCGAGTAGACAAC	GGAGCCTACCAGGTAAGAACA

## Data Availability

The original contributions presented in this study are included in the article. Further inquiries can be directed to the corresponding author.

## Abbreviations

The following abbreviations are used in this manuscript:

AGEs	Advanced Glycation End-Products
AKT	Protein kinase B
AMPK	AMP-activated protein kinase
ANOVA	Analysis of Variance
BAX	BCL2-associated X protein
BCA	Bicinchoninic Acid
BCL2	B-cell lymphoma 2
BD	Becton Dickinson
CASP3	Caspase-3
cDNA	Complementary DNA
Ct	Cycle threshold
ΔΔCt	Delta–delta Ct method
Ctl	Control
DMEM	Dulbecco’s Modified Eagle Medium
DMSO	Dimethyl sulfoxide
ECL	Enhanced chemiluminescence
EdU	5-Ethynyl-2′-deoxyuridine
EDTA	Ethylenediaminetetraacetic acid
EA.hy926	Human endothelial cell line
eNOS (NOS3)	Endothelial nitric oxide synthase (gene NOS3)
ERK1/2	Extracellular signal-regulated kinase 1/2
FBS	Fetal bovine serum
GAPDH	Glyceraldehyde-3-phosphate dehydrogenase
GPX4	Glutathione peroxidase 4
HIF-1	Hypoxia-inducible factor-1
HO-1 (HMOX1)	Heme oxygenase-1 (gene HMOX1)
HRP	Horseradish peroxidase
KEAP1	Kelch-like ECH-associated protein 1
MAPK	Mitogen-activated protein kinase
Matrigel	Basement membrane matrix
MGO	Methylglyoxal
MMP	Matrix metalloproteinase
mRNA	Messenger RNA
mTOR	Mechanistic target of rapamycin
mTORC1	Mechanistic target of rapamycin complex 1
NO	Nitric oxide
NQO1	NAD(P)H quinone dehydrogenase 1
NRF2	Nuclear factor erythroid 2-related factor 2
NP-40	Nonidet P-40
Oe	SESN2 overexpression
PBS	Phosphate-buffered saline
PCR	Polymerase chain reaction
PI3K	Phosphoinositide 3-kinase
PVDF	Polyvinylidene difluoride
qPCR	Quantitative PCR
qRT-PCR	Quantitative reverse-transcription PCR
RFU	Relative fluorescence units
RIPA	Radioimmunoprecipitation assay
SDS-PAGE	Sodium dodecyl sulfate–polyacrylamide gel electrophoresis
SESN2	Sestrin2
siRNA	Small interfering RNA
Si	SESN2 silencing
TBST	Tris-buffered saline with Tween-20
ULK1	Unc-51-like kinase 1
VEGFA	Vascular endothelial growth factor A
VEGFC	Vascular endothelial growth factor C
VEGFR2	Vascular endothelial growth factor receptor 2
